# A trade-off between shelf-life mitigation and nutritional retention: a persistent challenge in pearl millet

**DOI:** 10.3389/fnut.2026.1864573

**Published:** 2026-09-07

**Authors:** Nisha Singh, Kinjalben Suthar

**Affiliations:** Gujarat Biotechnology University (GBU), Gandhinagar, India

**Keywords:** lipases, lipid oxidation, omics, pearl millet, rancidity, shelf life

## Abstract

Pearl millet serves as a vital cereal crop for ensuring food and nutritional security, particularly in arid and semi-arid regions known as “Grains of God,” due to its remarkable tolerance to drought and other adverse environmental conditions. In addition to its climate resilience, pearl millet possesses superior nutritional quality, being enriched with essential amino acids, dietary fiber, iron, zinc, and several health-promoting bioactive compounds, thereby making it an important component of sustainable and nutritious food systems. However, a major limitation associated with the commercial utilization of pearl millet is its susceptibility to rancidity during storage. Rancidity primarily results from the oxidative degradation of lipids, leading to the formation of free fatty acids, hydroperoxides, aldehydes, and other volatile compounds responsible for undesirable off-flavors and reduced sensory acceptability. This deterioration is largely attributed to enzymatic reactions involving polyunsaturated fatty acids, which adversely affect the shelf life and consumer preference of pearl millet-based products. In addition to the limited long-term efficacy of conventional stabilization approaches, these strategies are often associated with nutrient loss, deterioration of quality attributes, reduced scalability, and increased processing costs. Recent advances in “Omics” approaches, such as genomics, transcriptomics, lipidomic, and metabolomics, have substantially enhanced our understanding of the molecular mechanisms underlying rancidity development in pearl millet. Several candidate genes, including *PgTAGLip1*, *PgTAGLip2*, *LOX*, phospholipases, and lipid metabolism regulators, have been implicated in flour stabilization and lipid degradation pathways. Nevertheless, significant challenges remain, including the effective integration of multi-omics datasets, the lack of standardized phenotyping protocols for storage-related traits, and difficulties in temporal transcriptomic analyses due to RNA degradation during flour storage. This review provides a comprehensive synthesis of the current biochemical, nutritional, molecular, and omics-based insights into pearl millet rancidity, with particular emphasis on future breeding strategies aimed at developing varieties with enhanced storage stability and improved end-use quality.

## Graphical Abstract

**Figure:**
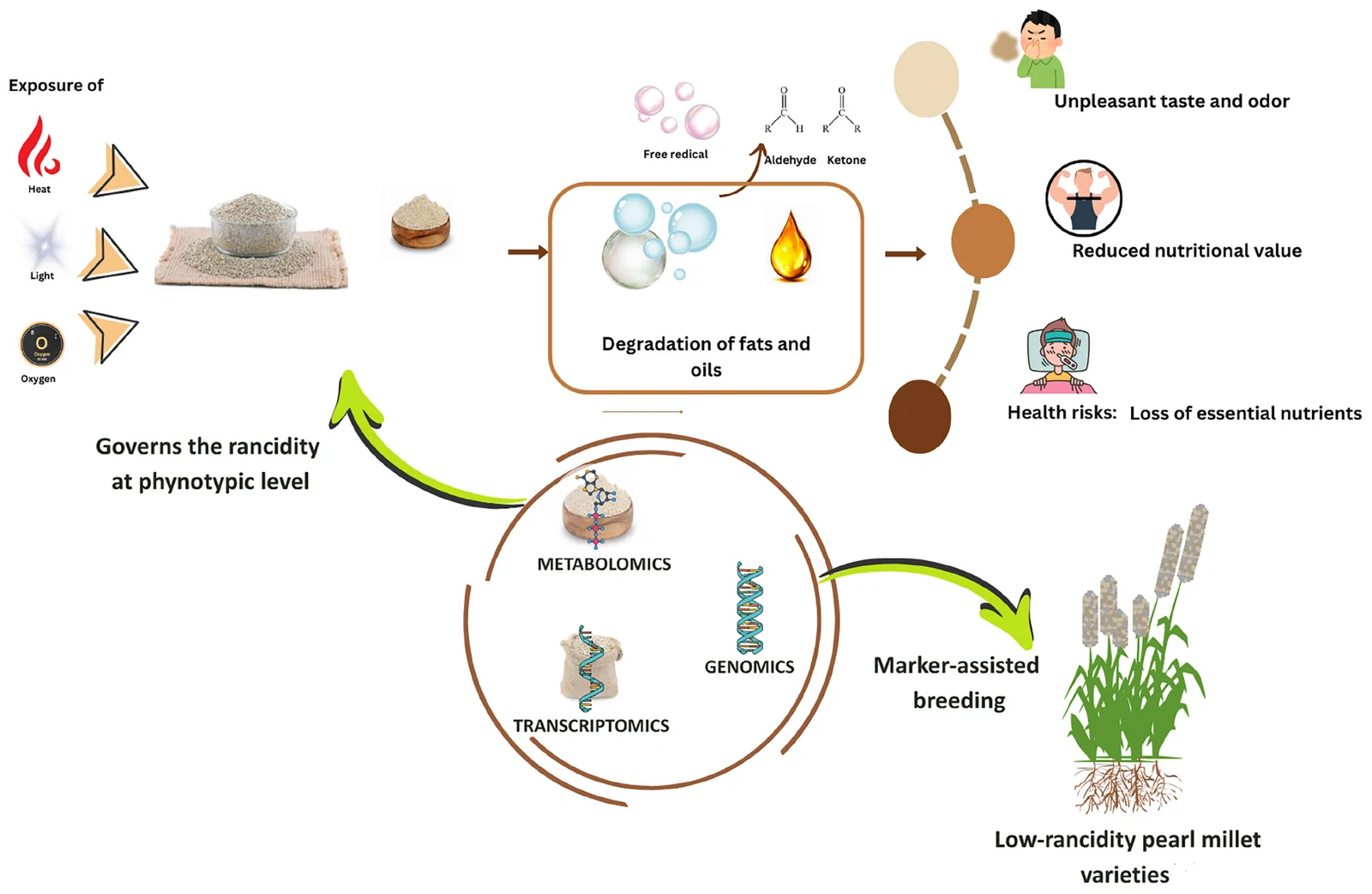
Environmental exposure and lipid degradation are the driving factors of rancidity progression in pearl millet. This will lead to the formation of volatile oxidation products, off tastes nutritional losses. Integrated multi-omics technologies such as metabolomics, transcriptomics and genomics can identify the molecular regulators of rancidity and support marker-assisted breeding by identifying molecular regulators of rancidity.

## Highlights

- Rapid rancidity limits the longevity and commercialization of pearl millet
- Lipases, LOX, and POX drive enzymatic lipid degradation post-milling
- High PUFA content increases susceptibility to oxidative deterioration
- Processing methods delay rancidity but compromise nutritional quality
- Omics approaches enable identification of genetic regulators of rancidity
- Integrated strategies are needed for sustainable shelf-life improvement.

## 1 Introduction

Millets are hardy crops that have the potential to thrive under adverse environmental conditions (1). Millets are members of the Poaceae family with unique nutritional benefits. Among them pearl millet (*Pennisetum glaucum* L.) is a staple food for many people and a good source of feed and fodder for livestock, especially in arid and semi-arid regions (2). Being a C4 plant, with a superior nutritional profile abundant with essential amino acids, iron (Fe), zinc (Zn), dietary fibers, and bioactive compounds, makes it a strategic crop for addressing food and nutritional security under escalating climate change (3, 4, 5). Due to its adaptability and nutritional abundance, Pearl millet has become one of the most widely cultivated and consumed millet species in developing countries. Pearl millet accounts for approximately 75% of the global millet cultivation area (4). It is mainly cultivated in India, Nigeria, Niger, China, Sudan, Ethiopia, Mali, and other South Asian countries. However, India is dominating pearl millet production globally by 38.4%. Between 2007 and 2019, the area of global millet cultivation reduced from 35 to 32 million hectares, but productivity increased significantly in Asia from 1100 kg/ha to 1292 kg/ha (4). Global millet productivity increased by 36%, from 575 kg/ha in 1961 to 900 kg/ha in 2018, highlighting continued improvements in millet production efficiency despite reductions in cultivated area (6). The present trend of developing sustainable food production systems globally has increased the interest in developing pearl millet-based functional foods.

Despite all these nutritive advantages, the market acceptance and commercialization of pearl millet-based foods are still limited due to the rapid onset of rancidity in milled flour. Pearl millet flour exhibits poor storage stability and develops undesirable off-flavors and odors within a short duration under ambient storage conditions, significantly reducing shelf life and marketability (7, 8). The rancidity of pearl millet flour is quite a complex phenomenon and occurs due to correlation between the lipid content, endogenous enzymatic activity, grain composition, and external conditions for storage. The grain contains relatively high concentrations of polyunsaturated fatty acids (PUFAs), particularly linoleic and linolenic acids, which are highly susceptible to hydrolytic and oxidative degradation (9). The structure of intact grain keeps lipids and lipid-degrading enzymes separate, which become active after milling and accelerate the formation of volatile oxidation products, which generate off-flavors and result in quality deterioration (8, 10, 11). Fe and copper (Cu) function as pro-oxidants by accelerating the formation of free radicals, which fastens the lipid peroxidation and causes food deterioration (12, 13).

Post-harvest processing methods have been developed to suppress lipid-degrading enzymes and prolong flour shelf life (14–16). However, these methods are effective for a limited time period and offer few trade-offs in terms of nutrient retention, cost, acceptability, scalability, and feasibility for smallholder farming systems (9, 17). Consistent reliance on post-harvest processing methods has not provided an extended and sustainable solution. In contrast, the improvement efforts in pearl millet have mostly focused on yield increment, drought, disease resistance, and micronutrient content. The development of high-quality genome assemblies, high-density SNP (Single Nucleotide Polymorphism) maps, QTL (Quantitative Trait Loci) analysis, and multi-omics integration in pearl millet has paved a new path in the deeper understanding of the molecular mechanisms of rancidity and shelf-life stability (18–20). These studies have highlighted that rancidity progression is not only post-harvest but also a genetically regulated problem. However, limited information is available, and there is significant scope to see a holistic rancidity mechanism via integrating genetic, biochemical, sensory, and metabolomics data along with single-cell omics technology to increase shelf life.

The present review provide a holistic view on the mechanism of rancidity development from biochemical, molecular, and technological dimensions, highlighting the trade-off between shelf-life improvement and nutritional retention. The review highlights the enzymatic and oxidative mechanisms underlying rancidity, the limitations of current mitigation strategies, and the emerging roles of genomics, transcriptomics, lipidomics, and metabolomics in developing storage-stable pearl millet cultivars. Furthermore, the review identifies key research bottlenecks and future priorities required for integrating post-harvest technology with omics-assisted breeding approaches to improve the commercial viability and nutritional sustainability of pearl millet-based food systems.

## 2 Biochemical and molecular mechanisms of rancidity progression in pearl millet

Lipid abundance is the primary variable, making pearl millet susceptible to rancidity. Grains contain around 5%–7% total lipids, which are primarily located in the germ and aleurone layers, which contain active enzymes governing lipid metabolism (21). Although this lipid abundance contributes positively to nutritional quality and energy density, it also increases susceptibility to rapid post-milling deterioration. Pearl millet lipids are abundant in PUFA, specifically linoleic (C18:2) and linolenic (C18:3) acids. However, these fatty acids enhance the grain’s nutritional quality by improving cardiovascular health, while their multiple double bonds make them significantly vulnerable to enzymatic hydrolysis and oxidative damage (22, 23). The process of milling disrupts cellular integrity and exposes stored triacylglycerols (TAGs) to lipases, lipoxygenases (LOX), peroxidases (POX), and polyphenol oxidases (PPO). As a result, it shows a limited equilibrium between nutritional appeal and chemical instability (24, 25).

The onset of rancidity progression is due to the activation of hydrolytic and oxidative pathways after milling. Endogenous lipases are critical accelerators for catalytic hydrolysis during hydrolytic rancidity, which catalyze the breakdown of triacylglycerols (TAGs) into diacylglycerols (DAGs), monoacylglycerols (MAGs), and free fatty acids (FFAs), which are highly concentrated in the aleurone layer, pericarp, and germ of the grain (26). The abundance of unsaturated fatty acids (USFAs) makes pearl millet susceptible to rancidity, further accelerating the breakdown of FFAs (7, 8). The liberation of FFAs is a critical biochemical trigger, as it not only contributes directly to soapy and bitter off-flavors but also generates substrates highly prone to oxidation. As a result, these FFAs undergo Lipoxygenase (LOX)- mediated oxidation, leading to the formation of secondary oxidation products, such as aldehydes, ketones, and alcohols (11, 27). Among these, hexanal, nonanal, and aldehyde have been proposed as chemical markers of rancidity progression (28, 29). Further, Peroxidase (POX) and polyphenol oxidase (PPO) enhance oxidative breakdown by generating free radicals and promoting oxidative polymerization during storage (10).

**FIGURE 1:**
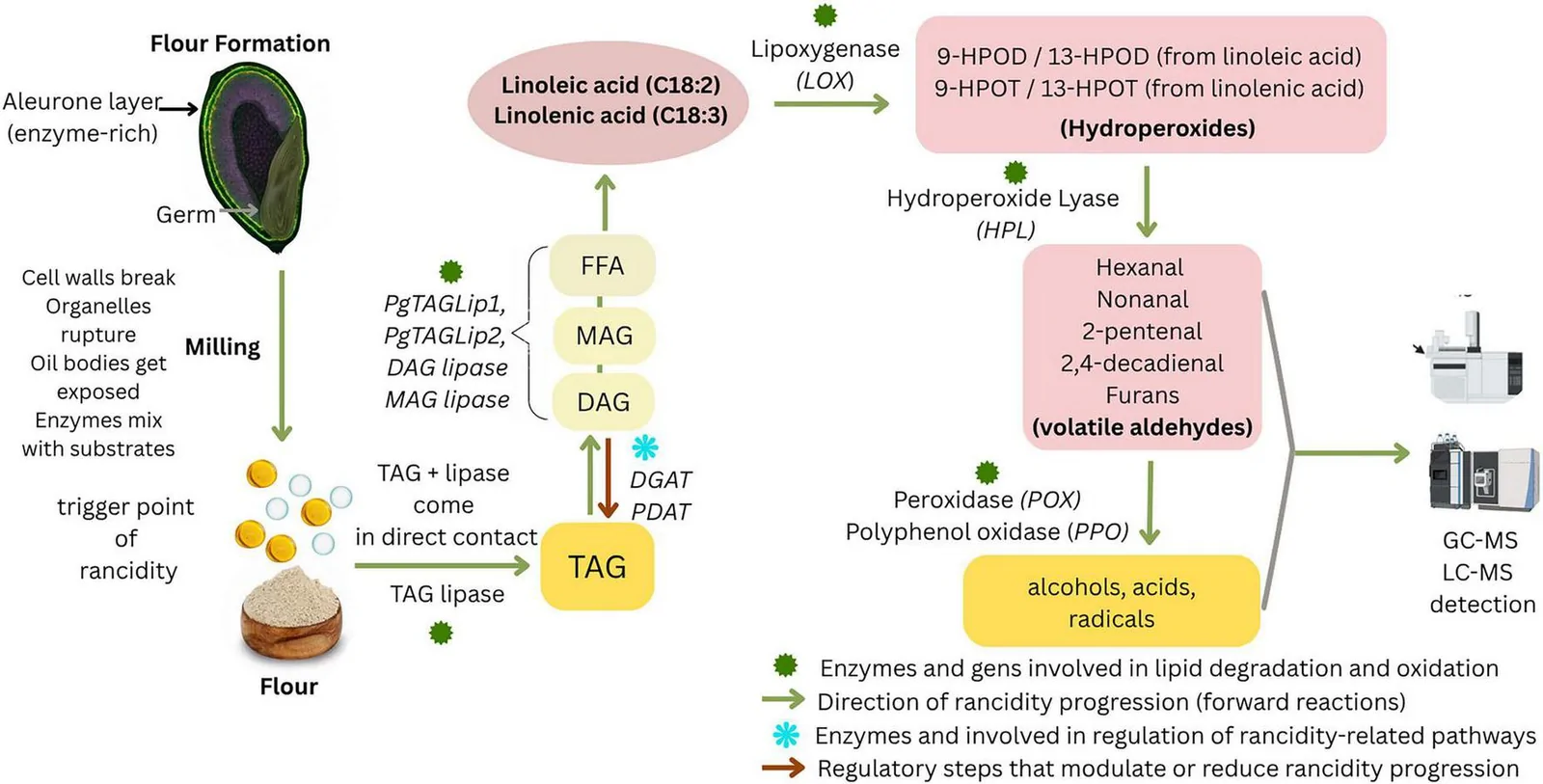
Pathway of rancidity development in pearl millet integrating lipid metabolism, gene regulation, and volatile compound formation. Rancidity affecting nutrition stability and consumer acceptability of pearl millet.

At the molecular level, genetic variations have been observed in lipid levels, enzyme activities, and rancidity formation among varieties of pearl millet, suggesting that flour stability is primarily genetically governed (8, 18). Mutations that result in loss of function in lipolytic enzymes such as *PgTAGLip1* and *PgTAGLip2* have been found, which gives strong support to the idea that, rather than relying solely on stabilization procedures after harvest, flour stability can be improved through molecular breeding and omics-based approaches (18, 20). The hypothetical pathway illustrating the overall rancidity progression mechanism, along with associated genes and enzymes, is presented in Figure 1 and in Table 1. The genes and their role in rancidity mitigation are also illustrated. Recent discoveries have shifted the focus of current research toward using biochemistry, genomics, and multi-omics data to develop storage-stable varieties of pearl millet with minimal nutritional losses. The hypothetical mechanism of the rancidity progression pathway has been illustrated in Figure 1.

**TABLE 1:** List of genes involved in lipid degradation, rancidity development, and storage stability in pearl millet and their functional roles.

Gene / protein	Functional role in rancidity	Key findings	Omics/experimental approach	References
*LOX-1*	Lipid oxidation and volatile formation	RNAi-mediated silencing in rice reduced rancid volatile compounds, supporting analogous role in pearl millet	Functional genomics and RNAi validation	Roy Chowdhury et al. (46)
*PgTAGLip1*	Lipase-mediated hydrolytic rancidity	Higher lipase activity associated with high-rancidity cultivars	Biochemical assays and storage studies	Goswami et al. (8)
*POX family (Class III)*	Oxidation of FFAs and hydroperoxides; free radical generation	Increased POX activity observed in stored flour samples; implicated in oxidative deterioration	Enzyme assays and genomic surveys	Goswami et al. (8)
*PgTAGLip1*	Triacylglycerol (TAG) hydrolysis; TAG → DAG + FFA	Loss-of-function alleles identified in low-rancidity genotypes; truncated protein associated with improved flour stability and reduced FFA accumulation	Functional genomics, gene cloning, mutant analysis, transcriptomics	Aher et al. (18)
*PgTAGLip2*	TAG → DAG + FFA; complementary TAG lipase activity	Loss-of-function mutation associated with delayed rancidity development and reduced lipase activity	Functional genomics and mutant characterization	Aher et al. (18)
*PgPLDα1-1*	Phospholipid degradation; phospholipid → FFA	Grain-specific candidate gene associated with rancidity-related lipid metabolism	Genomics and transcriptomics	Moin et al. (19)
*PgPLDα1-5*	Phospholipid hydrolysis and membrane lipid turnover	Identified as grain-expressed candidate associated with storage instability	Genomics and transcriptomics	Moin et al. (19)
*PgPLDδ1-7a*	Phospholipase D-mediated lipid degradation	Candidate gene potentially involved in oxidative deterioration during storage	Comparative genomics	Moin et al. (19)
*PgPLDδ1-2a*	Phospholipid degradation and FFA generation	Grain-specific phospholipase candidate linked with rancidity pathways	Comparative genomics	Moin et al. (19)
*PgPLA1-II-1a*	Phospholipase A1 activity; PL → lysophospholipid + FFA	Homologous to rice PLA1; proposed contributor to membrane lipid degradation	Genomics and homology-based annotation	Moin et al. (19)
*PgTAGLip1*	TAG degradation and rancidity progression	Differential expression associated with rancidity susceptibility and volatile accumulation during storage	Transcriptomics and metabolomics integration	Kumar et al. (36)
*MAGL (MZ590564)*	Monoacylglycerol hydrolysis; MAG → FFA + glycerol	Expression positively correlated with rancidity markers and FFA accumulation; conserved catalytic motif identified	Gene cloning, transcriptomics, enzyme characterization	Kumar et al. (20)
*TAGL (MZ590565)*	TAG hydrolysis and FFA release	Differential expression linked to storage instability and rancidity progression	Gene cloning and transcript profiling	Kumar et al. (20)
*LOX-1 (OQ184873)*	Oxidation of ω-6 PUFAs to hydroperoxides	Upregulated expression observed in high-rancidity cultivars; associated with volatile aldehyde formation	Transcriptomics, comparative expression analysis	Kumar et al. (37)
*LOX-6 (OQ184874)*	13-LOX-mediated PUFA oxidation	Presumed involvement in lipid peroxidation and hydroperoxide generation during storage	Gene cloning and comparative functional analysis	Kumar et al. (37)
*POX family (Class III)*	Oxidative stress and peroxide metabolism	Large POX gene family identified in pearl millet genome; linked with oxidative deterioration pathways	Genomics and comparative gene family analysis	Kumar et al. (37)
*PgPDAT1*	TAG remodeling and acyl transfer	Overexpressed in high-rancidity genotype; proposed target for genome editing	Transcriptomics and comparative genomics	Moin et al. (47)
*PgDGAT1*	TAG biosynthesis and lipid accumulation	Grain-specific overexpression associated with rancidity-prone genotype	Transcriptomics and comparative genomics	Moin et al. (47)
*PgDREB2A*	Stress-responsive transcription factor linked to lipid metabolism	Proposed regulator of fatty acid accumulation and stress-induced lipid signaling	Transcriptomics and stress-response analysis	Moin et al. (47)

Apart from biochemical and molecular factors, environmental conditions also regulate the onset of rancidity, including moisture, temperature, and oxygen. An increase in average moisture level increases enzyme mobility and catalytic efficiency by enhancing lipase-mediated hydrolysis and LOX-driven oxidation (10). Although very low moisture levels can reduce the enzyme activity, normal storage conditions maintain enough moisture to sustain enzymatic degradation. Humidity fluctuation during storage can lead to intermittent enzyme activation and oxidative stress, further accelerating the rancidity (8). High storage temperature exacerbates oxidation kinetics and hydroperoxide decomposition (10, 30). Oxygen availability is another key factor; atmospheric oxygen promotes the action of lipoxygenase while maintaining the autooxidation chain reaction. Oxygen-diffusing packaging arrangement limits the shelf life compared to vacuum-sealed or inert atmospheric storage (31). Natural antioxidants such as phenolic compounds, tocopherols, and flavonoids delay lipid oxidation by scavenging free radicals. However, milling disrupts antioxidant localization and accelerates their depletion, thereby reducing protective capacity during storage (17).

## 3 Rancidity affecting nutritional stability and consumer acceptability of pearl millet

The nutrient-dense composition of pearl millet contributes significantly to its nutritional and health benefits. However, the presence of phytates and tannins reduces the body’s ability to absorb iron and zinc, allowing only a small portion (10%–15%) to be utilized. During storage, rancidity can further lower the nutritional quality of pearl millet by degrading nutrients and reducing their availability (32, 33). During autooxidation, naturally occurring antioxidants and vitamins are rapidly depleted, leading to the loss of nutritionally important compounds (34). Antioxidants, which are found naturally (such as vitamin E and phenolic acids), when exposed to oxidized compounds (such as FFAs and polymers), may bind minerals (32). Moreover, oxidized lipid products, such as FFAs and lipid polymers, may interact with minerals, thereby reducing their bioavailability (35). Hence, the rich content of vitamins A and B, along with antioxidants such as ferulic and coumaric acids, is highly susceptible to oxidative degradation during storage-induced rancidity (32). The result will be flour with fewer antioxidants and heat-labile vitamins, which will not be able to absorb minerals as well as fresh flour.

The nutritional value of pearl millet flour deteriorates as rancidity increases. However, as shown in Figure 1, after post-milling, lipase- and LOX-mediated pathways lead to the formation of FFAs, hydroperoxides, and volatile aldehydes such as hexanal, nonanal, and 2-octenal (20). These substances exhibit good correlation with the increasing amounts of peroxide and acid values and are responsible for the development of bitterness and “paint-like” off-flavor after 5 to 10 days of storage (33, 36). Recently, one such correlation was reported where the rancid compounds (hexanal, nonanal, FFAs) increased in a parallel manner with peroxide value (PV) and acid value (AV), high ratio of AV/PV with high amounts of volatile aldehydes observed in susceptible genotypes like hybrid Pusa-1201 in comparison to landraces like Chadhi Bajri at same conditions (33, 37). Interestingly, the highest levels of peroxidised lipids were observed on the tenth day in many studies (32). After that, the flour is generally unpalatable.

The sensory spoilage of flour is linked to chemical signs of rancidity, namely hydroperoxides and aldehydes (LOX reactions), resulting in PV value increase and formation of FFAs (lipase reactions), leading to increased AV (29); Under usual conditions, the value of peroxide and acid values tends to double in just a week. In one experiment, it was noted that mousy odor or bitterness was detected and that PV and AV doubled within just 10 days (32). Considering the sensitivity of volatiles in ppb to Miller, the mere presence of the oxidation process produces such a smell. This is because, as noted by Hosseini Bai et al. (38), the panel members rejected the flour due to the accumulation of hexanal and nonanal caused by the storage period (20). Based on actual practice, when the values of FFA and PV reach 1–2 mg KOH/g and 0.5–1.0 meq O2/kg, respectively, consumers are expected to reject.

## 4 Omics Insights into the Development of Rancidity in Pearl Millet

Genotypic variation primarily influences the onset of rancidity in pearl millet; some genotypes may inherently have higher levels of antioxidants, leading to reduced decay. One such genotype was Chadhi Bajri, which had higher levels of quercetin and rosmarinic acid than Pusa-1201, a hybrid, under similar storage conditions (37). In contrast, varieties with high yields or fortified genotypes may be at risk of spoilage; high-iron genotypes tend to have higher levels of anti-nutrients and lipids. Research has revealed that high-iron genotypes tend to accumulate higher levels of FFAs while under storage (a “double-edged sword” due to the nature of fortification); this occurred especially in the case of high-iron genotypes, which had higher AV/PV ratios than those with low iron (13).

These findings highlight the importance of using omics technologies and genome-wide association studies (GWAS) to better understand and address rancidity in pearl millet. Recent research has focused more on the genetic factors that influence rancidity rather than on post-harvest methods to prevent it. For example, studies have shown that mutations in the genes *PgTAGLip1* and *PgTAGLip2*, which encode enzymes involved in fat breakdown, can significantly reduce the formation of free fatty acids (FFAs). This helps slow down rancidity and extends the storage life of pearl millet seeds without negatively affecting plant growth (32). Such genetic information can be used in future omics-based breeding programs to develop pearl millet varieties with improved resistance to rancidity and better storage stability.

## 5 Current strategies to mitigate rancidity: benefits and limitations

There are several ways to slow rancidity, using either physical or chemical methods. Significant efforts have been made to employ post-harvest processing approaches, including conventional and state-of-the-art methods, to delay the onset of rancidity in pearl millet. While attempts to extend shelf life have generally focused on reducing moisture content, inactivating lipases, or preventing oxidation, some approaches have not overcome constraints such as nutrient loss, costly procedures, scale-up issues, and limited applicability in small-farming settings. Consequently, none of these measures has so far provided an effective, nutritionally sustainable solution to rancidity in pearl millet.

### 5.1 Traditional pre-processing methods to mitigate rancidity

**FIGURE 2:**
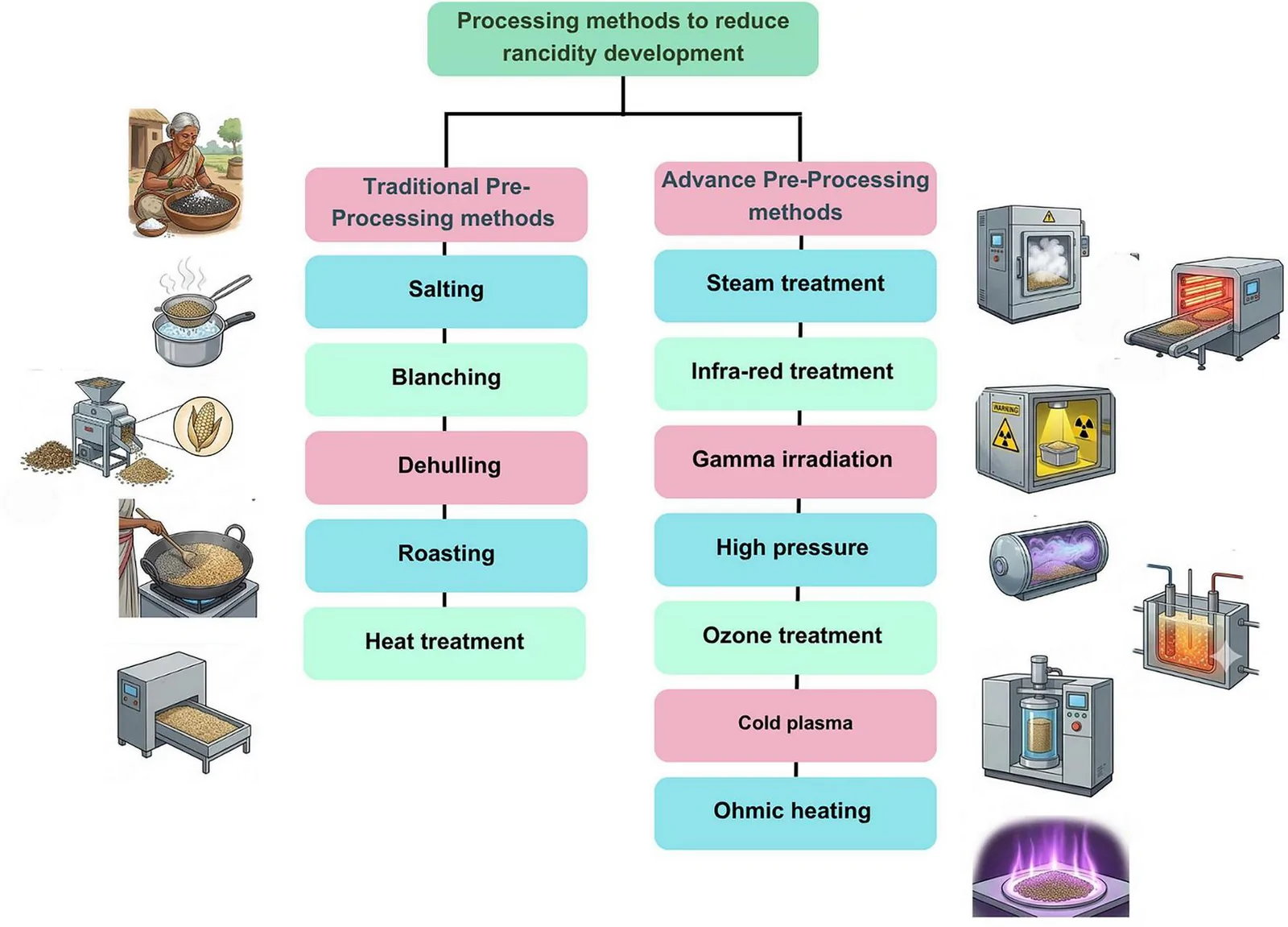
Overview of traditional and advanced processing strategies used to mitigate rancidity development and enhance the storage stability of pearl millet and its products.

Several traditional pre-processing methods, as shown in Figure 2, have been used to prevent rancidity and extend the shelf life of pearl millet flour. These methods aim to reduce lipid oxidation and microbial growth, the two primary causes of rancidity. Salting is the most common practice that adds a teaspoon of salt to pearl millet grain. This reduces water activity, inhibits microbial growth, and suppresses rancidity-causing enzymes by creating an unfavorable environment, making it both effective and convenient because of the availability of salts (39). Blanching, another effective method, involves submerging the grains in boiling water (98°C) at a 1:5 grain-to-water ratio for 30 min, followed by drying at 50 °C for 60 min. This process inactivates lipase, *LOX*, and *POX*, reducing anti-nutritional factors such as phytic acid and polyphenols, which extend the shelf life of flour (40). Dehulling removes the outer hull or bran layer from the grain and effectively reduces rancidity by eliminating lipid-rich components and the accompanying enzymes, thereby decreasing the likelihood of oxidation (39). Roasting is another traditional method that removes moisture and inactivates lipid-oxidizing enzymes, thereby preventing oxidation while enhancing the flavor and aroma of flour. However, proper temperature control is essential to avoid the production of undesirable compounds (39).

### 5.2 Advanced pre-processing methods to mitigate rancidity

To overcome the challenges of rancidity in pearl millet flour, several advanced pre-treatment methods have been employed, utilizing innovative technologies to inhibit lipid oxidation, inactivate rancidity-causing enzymes, and extend the shelf life of the flour. Steam treatment is one such method, in which pearl millet grains are exposed to superheated steam for up to 90 s. This effectively inactivates enzymes such as *lipase* and *LOX*, thereby reducing rancidity and improving the storage stability of flour (39). Infrared treatment uses near-infrared radiation applied to hydrated pearl millet grains or flour, generating heat that reduces enzyme activity and moisture content, effectively lowering the potential for lipid oxidation while preserving the color and texture of the flour (15). Gamma irradiation involves exposing the flour to gamma rays at doses between 1.5 and 5.0 KG, reducing microbial load, inactivating rancidity-promoting enzymes, and extending shelf life for up to 3 months under ambient conditions (16). High-pressure processing subjects grains to elevated pressure, inactivates enzymes, and reduces microbial activity without affecting the nutritional quality of flour (15). Similarly, ozone treatment exposes pearl millet to ozone gas. This powerful oxidizing agent reduces microbial contamination and inactivates enzymes, thereby delaying rancidity and extending the shelf life of the flour (39). Cold plasma technology employs ionized gas at room temperature to deactivate enzymes, reduce lipid oxidation, and improve nutrient bioavailability without compromising flour quality (39). Finally, ohmic heating passes an electric current through the grain or flour, generating internal heat that denatures enzymes responsible for lipid oxidation while preserving the nutritional and sensory properties of the flour (15). Modern processing technologies, such as oxidation kinetics modeling, biochemical monitoring of rancidity indicators, including PV, AV, FFAs, and lipase activity, have expedited storage research (41). Emergence of artificial intelligence (AI) and machine learning (ML) in pearl millet post-harvest models is using Artificial Neural Networks (ANN), Support Vector Regression (SVR), Gradient Boosting, and Long Short-Term Memory (LSTM). These networks are being utilized to predict shelf life and rate of rancidity progression based on storage and biochemical data. In accordance with recent findings, ANN models excelled traditional statistical techniques in predicting the shelf life of pearl millet grain with very high accuracy (R^2^ = 0.9847–0.9969) (42). Similarly, deep learning models like LSTM-based models have been applied for detecting flour stability using rancidity and nutritional indicators under different storage conditions (43).

These advanced methods provide effective strategies for addressing rancidity while maintaining the quality and nutritional value of pearl millet flour. Pearl millet’s nutritional qualities, ability to scavenge free radicals, and taste have been enhanced by germination, fermentation, high-pressure ultrasonication, irradiation, pulsed light, and cold plasma; on the other hand, the levels of micronutrients, polyphenols, and dietary fiber have been affected by severe dehulling, milling, and polishing (16). In contrast, thermal processes such as roasting, steaming, and infrared heating help fight rancidity but lead to the loss of water-soluble vitamins and other nutritious substances in the food; besides, lipases and LOX enzymes are deactivated, yet they still help preserve the food for about 2–3 months. Like thermal methods, fermentation has been useful in reducing rancidity due to decreased lipase and LOX enzyme activity, as well as PV and AV levels [with ≈3.6% fat in flour in (Damirga) fermentation compared to unfermented flour with high fat levels] (44). Unfortunately, the vitamin and phytonutrient content is lost during 10%–30% of the fermentation or processing of the flour. For instance, Damirga leads to increased levels of carbohydrate but decreases levels of fats (44). Nevertheless, most stabilization methods were only capable of providing a temporary fix for the problem and focused on addressing issues related to the storage of pearl millet after harvesting, rather than the chemistry and genetics underlying the development of rancidity. Most stabilization methods are expensive and impractical to use in the smallholder farmers’ setting, while at the same time posing challenges in terms of micronutrient retention, antioxidant content, and taste (17, 41, 45).

## 6 Genetic and molecular regulation of rancidity in pearl millet

The onset of pearl millet rancidity has long been linked with their storage after harvest, but contemporary research indicates that genetics regulating lipid metabolic pathways and the process of lipid oxidation are key players in flour stability. The genotype differences in terms of lipid composition, lipase activity, FFA levels, and aldehyde accumulation indicated that susceptibility to rancidity was partly genetic (8, 18). It is clear from comparative studies that the rate of TAG hydrolysis, the level of lipid oxidation, and the production of aldehydes such as hexanal and nonanal were much lower in low-rancidity genotypes than in high-rancidity ones (18). Transcriptomic studies in pearl millet and other rancidity-prone cereals highlight that genes encoding lipases, LOXs, and other related oxidative enzymes are functionally regulated and are susceptible to post-harvest triggers such as milling, storage temperature, and moisture. Rapid FFA accumulation and rapid off-flavors development rate are linked to increased expression of lipase and LOX gene families (15). Furthermore, significant genotype diversity in these genes’ baseline and inducible expression implies a key hereditary component driving rancidity susceptibility. However, transcriptomic analysis focused on post-milling storage stages in pearl millet still remains scarce. There is a major understanding gap regarding how transcriptional reprogramming during storage influences lipid deterioration, as most current datasets rely on grain development or abiotic stress responses. The vitality of time-resolved transcriptome profiling or single-cell transcriptome for feasible storage conditions is highlighted by this problem. Moreover, transcriptomic analysis of flour during different time points remains a technical challenge due to rapid RNA degradation during milling and further storage. In processed flour samples, only a limited amount of intact RNA remains detectable, resulting in frequently low RNA integrity numbers (RIN). Thus, degraded RNA quality adversely affects the library preparation, sequencing efficiency, and read accuracy. Therefore, it is challenging to reliably profile gene expression across different storage time periods, and transcriptomic methods may give a partial understanding of the biochemical alterations associated with storage.

Lipidomics is a powerful technique for identifying the molecular species that trigger rancidity. LC-MS and GC-MS techniques reveal that rancidity is associated with the accumulation of free fatty acids, lysophospholipids, and oxidized lipid species, as well as the progressive depletion of phospholipids and triglycerides (13, 17). Hydroperoxides and secondary volatile chemicals arise when PUFAs, especially linoleic acid and linolenic acids, act as a primary substrate for enzymatic oxidation. Additionally, cultivar-specific variations in lipid composition were also identified by lipidomic profiling, illustrating that intrinsic variations in fatty acid profiles may affect the oxidation susceptibility (48). However, the predictive potential of lipidomic studies for breeding applications is limited due to frequent analytical limitations and a lack of integration with enzymatic activity or gene expression data. However, metabolomic analysis provides direct links between biochemical decay and sensory perception of rancidity. Aldehyde, alcohol, ketone and short-chain fatty acids derived from lipid oxidation aggregate rapidly during storage and are also responsible for significant off-odors (28, 30). Secondary metabolites such as hexanal, nonanal, and aldehyde have been proposed as chemical markers of rancidity progression. Despite these advancements, metabolomic studies in pearl millet often lack quantitative thresholds that correlate metabolite abundance with consumer acceptability. Furthermore, few studies link sensory evaluation with metabolite profiling, limiting translation quality control or breeding metrics.

Nevertheless, the integration of genomics, transcriptomics, metabolomics, and lipidomics is creating new opportunities for developing storage-stable pearl millet cultivars with minimal nutritional compromise. Advances in genome-wide association studies (GWAS), quantitative trait locus (QTL) mapping, transcriptome-wide association studies (TWAS), and high-throughput sequencing technologies are enabling the identification of regulatory networks associated with lipid degradation and oxidative stress responses (47, 49). Emerging genome-editing approaches, such as Clustered Regularly Interspaced Short Palindromic Repeats (CRISPR/Cas-mediated) modification of lipase and LOX-related genes, may further accelerate the development of low-rancidity pearl millet lines with improved flour shelf life. Therefore, future breeding strategies should focus on integrating molecular understanding of rancidity with nutritional quality, sensory acceptance, and storage performance to achieve commercially viable, nutritionally sustainable pearl millet-based food systems.

## 7 Integrated multi-omics frameworks for rancidity control

### 7.1 Research gaps, challenges, and future priorities

Despite considerable progress in understanding pearl millet rancidity, several critical gaps remain that limit the development of effective, sustainable solutions to improve flour shelf life. Most current studies focus primarily on short-term biochemical changes or on individual enzymes, such as lipases and lipoxygenases (LOX), while the broader regulatory interactions among lipid metabolism, oxidative stress responses, storage conditions, and sensory deterioration remain poorly understood. Furthermore, many studies are conducted under controlled laboratory conditions using limited genotype panels, making it difficult to extrapolate findings to diverse production systems, climatic conditions, and real-market storage environments. One of the major bottlenecks in rancidity research is the lack of standardized phenotyping protocols for evaluating storage stability. Parameters such as free fatty acid accumulation, volatile compound formation, enzyme activity, and sensory rejection are often measured independently using different methodologies, leading to poor comparability across studies. In addition, the relationship between biochemical rancidity markers and actual consumer perception remains poorly established. Many volatile metabolites associated with lipid oxidation have been identified; however, the threshold levels that determine sensory rejection and nutritional deterioration remain unclear. Consequently, there is an urgent need for integrated studies combining metabolomic, lipidomic, sensory science, and nutritional analysis to develop reliable predictive biomarkers of flour stability.

Another important challenge is the limited integration of rancidity-associated traits into pearl millet breeding programs. Although candidate genes such as *PgTAGLip1* and *PgTAGLip2* have been identified, their validation across genetically diverse germplasm and environmental conditions remains inadequate (18). In addition, the possible physiological trade-offs associated with suppressing lipid metabolism pathways, including effects on seed germination, membrane integrity, and stress adaptation, require further investigation. Current breeding efforts are also constrained by insufficient high-throughput phenotyping platforms and the absence of validated molecular markers for the routine selection of low-rancidity genotypes.

Although individual omics platforms provide insights into specific aspects of rancidity progression, the highly interconnected nature of lipid degradation requires integrated multi-omics approaches that can link genotype, gene expression, metabolic changes, and sensory outcomes. Advances in genome-wide association studies (GWAS), quantitative trait locus (QTL) mapping, transcriptome-wide association studies (TWAS), and high-throughput sequencing technologies are enabling the identification of regulatory networks associated with lipid degradation and oxidative stress responses (47, 49). Emerging genome-editing approaches such as CRISPR/Cas-mediated modification of lipase and LOX-related genes may further accelerate the development of low-rancidity pearl millet lines with improved flour shelf life. Therefore, future breeding strategies should focus on integrating molecular understanding of rancidity with nutritional quality, sensory acceptance, and storage performance to achieve commercially viable, nutritionally sustainable pearl millet-based food systems. Recent advances in machine-learning-assisted data integration are providing new opportunities to accelerate the identification of key molecular determinants of flour stability (47, 49). Future research should therefore prioritize integrated multi-omics and single-cell omics approaches capable of linking genomic variation, transcriptional regulation, metabolic signatures, and sensory outcomes associated with rancidity progression. Long-term storage studies involving diverse germplasm collections and realistic environmental conditions are essential for identifying stable low-rancidity traits suitable for commercial cultivation. Moreover, integrating genome editing, artificial intelligence-assisted data analysis, predictive modeling, and marker-assisted breeding could substantially accelerate the development of pearl millet cultivars with improved storage stability and nutritional retention. Importantly, future strategies should not focus solely on extending shelf life but also on preserving micronutrient bioavailability, sensory quality, and consumer acceptance to ensure the successful commercialization of pearl millet-based functional foods within climate-resilient, nutrition-sensitive food systems.

## 8 Conclusions and outlook

In pearl millet, rancidity is the major bottleneck to the widespread utilization in the modern food system, despite being nutritionally rich. Compared to other cereals, the rapid progression of lipid degradation in pearl millet is governed by high lipid content, abundance of PUFAs, and increased activity of lipid-degrading enzymes. Conventional processing-based techniques are only partially effective at addressing symptoms rather than the root cause, thereby compromising nutritional quality. This review underlines that a paradigm shift from post-harvest management to inherent grain stability will be vital for long-term rancidity prevention. Recent technological advances in the omics technology, such as genomics, transcriptomics, metabolomics and lipidomics, offer new information to establish the understanding of molecular determinants of rancidity and implement them into the upcoming breeding programs. However, the advancement of standardized phenotyping technologies and the integration of food chemistry and agricultural enhancement studies are important to achieve this goal. By integrating biochemical understanding with omics-driven breeding strategies, it is feasible to develop a pearl millet cultivar with nutritional excellence and reduced susceptibility to rancidity. These advances will help to rebrand the pearl millet for its benefits by enhancing consumer acceptance, market opportunities and will also address its role in sustainable and climate-resilient food systems.
